# 인공호흡기 관련 폐렴과 인공호흡기 관련 사건 감시체계의 비교 분석: 후향적 관찰 연구

**DOI:** 10.7586/jkbns.26.020

**Published:** 2026-08-25

**Authors:** Seo Jin Jeon, Jae Geum Ryu

**Affiliations:** 1 Neurological ICU, Dong-A University Hospital, Busan, Korea; 1 동아대학교병원 신경계중환자실; 2 College of Nursing, Dong-A University, Busan, Korea; 2 동아대학교 간호대학

**Keywords:** Pneumonia, ventilator-associated, Population surveillance, Intensive care units, Infection control, 인공호흡기 관련 폐렴, 인구집단 감시, 중환자실, 감염관리

## Abstract

**Purpose:**

Ventilator-associated pneumonia (VAP) surveillance has been widely used for infection surveillance in intensive care units (ICUs), but diagnostic subjectivity and a high monitoring burden remain major limitations. Ventilator-associated event (VAE) surveillance was introduced to improve objectivity and efficiency; however, few studies have directly compared these two surveillance systems. This study compared VAP and VAE surveillance by examining incidence, associated factors, and surveillance time.

**Methods:**

This retrospective observational study included patients aged ≥ 15 years who received mechanical ventilation for more than 2 days in adult ICUs of a tertiary hospital. VAP was defined according to Centers for Disease Control and Prevention/National Healthcare Safety Network (CDC/NHSN) criteria, and VAEs were identified and classified according to the 2025 CDC/NHSN definitions. Multivariable logistic regression analysis was performed to identify independently associated factors.

**Results:**

Among 942 patients, 31 VAP events and 84 VAEs were identified, corresponding to 2.9 and 8.0 events per 1,000 ventilator-days, respectively. Surveillance time was shorter for VAE than for VAP (mean, 0.7 vs. 2.5 minutes per patient). Prolonged mechanical ventilation was independently associated with both VAP and VAE, whereas corticosteroid use was associated with VAP and possible VAP. The proportion of ICU days with deep sedation, defined as a Richmond Agitation–Sedation Scale score ≤ −3, was associated with all VAE categories but not with VAP.

**Conclusion:**

VAE was associated with care process-related factors, whereas VAP with infection-related factors, suggesting that the two systems may provide complementary information as ICU surveillance metrics.

## 서론

### 1. 연구의 필요성

인공호흡기 관련 폐렴(ventilator-associated pneumonia, VAP)은 인공호흡기 적용 후 48시간 이후 발생하는 폐렴으로, 중환자실에서 발생한 삽입기구관련 감염 중 가장 중증도가 높은 것으로 알려져 있다[1,2]. 특히 VAP는 다른 삽입기구관련 감염에 비해 환자 예후에 미치는 영향이 크며, 최대 기여사망률이 13%에 이르고, 중환자실 재원기간을 약 12일 연장시키는 등 중요한 임상적 문제가 되고 있다[2]. 그러나 VAP는 흉부 방사선 소견, 임상적 증상 및 미생물학적 검사결과를 종합하여 진단하는 과정에서 감시자의 주관적인 판단이 개입되어 감시자간 편차가 발생하고 감시 결과의 타당도와 신뢰도가 낮아질 수 있다는 문제가 지속적으로 제기되어 왔다[3–5]. 국내 Kwak [4]은 질병관리청이 운영하는 전국 의료관련감염 감시체계(Korean Nosocomial Infections Surveillance System, KONIS)의 VAP 감시 타당도를 평가한 연구에서 대부분 민감도가 60%~70%로 낮았고, 연구 간 변동성은 커 일관성이 부족하다고 보고하였다. 미국의 Klompas 등[5] 또한 VAP 감시 결과가 의료기관 간 큰 차이를 보이며 실제 발생률을 과소평가한다는 점을 지적하였다.

VAP 진단의 주관성 개입 가능성과 이로 인해 과소평가되는 문제를 극복하기 위해 미국 질병통제예방센터Centers for Disease Control and Prevention, CDC)의 국가 의료관련 감시체계(National Healthcare Safety Network, NHSN)는 2013년 인공호흡기 관련 사건(ventilator-associated event, VAE) 감시체계를 도입하였다[5,6]. VAE는 VAP와 달리, 흡입산소분율(fraction of inspired oxygen, FiO_2_) 또는 호기말양압(positive end-expiratory pressure, PEEP)의 지속적 증가, 감염 징후, 미생물학적 검사 결과와 같은 정량적 지표를 사용하여 환자 상태를 평가하므로 주관성을 줄이고 객관성과 타당성을 높일 수 있는 것으로 평가된다[7,8]. VAE는 임상 상태의 변화에 따라 세 단계의 위계 구조를 가지며, 인공호흡기를 적용하면서 발생하는 환자 상태의 악화를 연속적인 궤적(trajectory)에 따라 감시한다[6]. 즉, 인공호흡기 관련 이상 상태(ventilator-associated condition, VAC)는 인공호흡기 설정의 지속적인 증가를 통해 환자 상태 악화를 반영하고, 인공호흡기 관련 감염 합병증(infection-related ventilator-associated complication, IVAC)은 이에 더하여 체온 또는 백혈구 수치의 변화와 항생제 사용이 동반된 경우를 포함하며, 가능성 있는 인공호흡기 관련 폐렴(possible ventilator-associated pneumonia, PVAP)은 여기에 미생물학적 근거가 추가된 경우를 의미한다[6,7]. VAE는 진단에 활용되는 지표들이 이미 의무기록에 기록되어 있어 이를 기반으로 감시를 수행할 수 있으며, 전자의무기록(electronic health record, EHR)을 활용하면 감시 과정의 효율성과 결과의 정확성을 높일 수 있을 것으로 보고된다[5,8–10].

이러한 VAE 감시의 장점을 바탕으로 기존 감시체계를 VAE로 대체할 수 있는지를 평가하기 위해 일치도뿐 아니라 위험요인을 함께 분석한 다각적 연구들이 수행되었다[10–13]. 이들 연구 결과는 VAP와 VAE가 동일한 임상 현상을 반영하기보다는 서로 다른 감시 개념에 기반한 체계임을 시사한다[11,12]. 국내 연구와 체계적 문헌고찰 및 메타분석 연구에서도 VAE는 VAP를 충분히 탐지하지 못하는 것으로 보고되었으며, 민감도는 약 40% 내외, 양성예측도 또한 25% 미만 수준으로 전반적으로 낮은 경향을 보였다[10,11]. 또한 VAP는 성별, 흡연 여부, 질병 중증도와 같은 수정이 어려운 환자 요인과 관련되는 반면, VAE는 깊은 진정, 높은 인공호흡기 설정, 수액 과다, 수혈 및 장기간 인공호흡기 사용과 같은 치료 과정에서의 수정 가능한 요인과 관련되었다[12,13].

이러한 이유로 중환자실의 인공호흡기 관련 폐렴 감염 감시체계의 선택과 활용에 대해 다양한 의견이 제시되고 있다. 유럽 질병예방통제센터(European Centre for Disease Prevention and Control)는 VAP가 임상적 폐렴 진단 및 치료 결정과 밀접하게 연관되어 있고 VAP와 VAE 간 감시 일치도가 낮다는 점을 근거로 VAP 진단체계의 유용성을 강조하였다[14]. 국내에서도 질병관리청이 VAE 감시체계의 적용 가능성을 평가하였으나 두 감시체계 간 낮은 일치도와 더불어 중환자실에서 EHR 활용을 위한 기반이 충분히 구축되어 있지 않은 점을 고려하여 VAP 중심의 감시체계를 유지하고 있다[10]. 그러나 VAE는 인공호흡기 적용 중 발생하는 상태 악화를 반영하여 환자의 예후 악화를 예측할 수 있는 유용한 지표로 보고되었다[8,15,16]. 유럽연합에서 수행된 성인 대상 VAE 연구(EUVAE study) [17]와 소아 대상 VAE 연구(EUVAE-kids study) [18]에서는 VAE가 사망률 및 재원기간 연장과 유의한 관련을 보였으며, He 등[15]의 5년간 대규모 감시연구에서도 VAE는 인공호흡기 사용기간 및 재원기간 연장, 병원 내 사망률 증가와 유의한 관련을 보였다.

국내에서는 질병관리청에서 VAE 감시체계에 대한 시범 연구가 수행된 적은 있으나[10], VAP와 VAE 감시 결과를 직접 비교하고 발생률과 관련 요인을 포괄적으로 탐색한 연구가 부족하여 두 감시체계의 발생률과 관련 요인 및 감시 부담 측면에서 종합적으로 비교한 근거는 제한적이다. 따라서 본 연구는 중환자실의 EHR 시스템이 잘 구축된 일 상급종합병원의 성인 중환자실을 대상으로 VAP와 VAE 감시체계의 특성을 비교 분석하고 두 감시체계의 활용방안을 모색해보고자 한다.

### 2. 연구의 목적

본 연구의 목적은 일 상급종합병원의 성인 중환자실을 대상으로 VAP와 VAE 감시체계를 적용하여 두 감시체계의 감시결과를 비교하고, 감시시간 차이를 확인하는 것이다. 구체적인 목적은 다음과 같다.

1) 성인 중환자실 환자에서 VAP와 VAE의 발생률을 비교한다.

2) 성인 중환자실 환자에서 VAP와 VAE의 관련 요인을 비교한다.

3) VAP 감시와 VAE 감시에 소요되는 감시시간을 비교한다.

## 연구 방법

### 1. 연구설계

본 연구는 성인 중환자실 환자를 대상으로 VAP와 VAE의 발생률, 관련 요인 및 감시시간을 비교한 후향적 관찰연구로, STROBE (Strengthening the Reporting of Observational Studies in Epidemiology) 지침에 따라 보고하였다.

### 2. 연구 대상

본 연구의 대상자는 2022년 1월 1일부터 2022년 12월 31일까지 부산에 위치한 일 상급종합병원의 성인 중환자실(4개 병동, 총 65병상)에 입실한 환자 중, 자정(00:00)을 기준으로 날짜를 계산하는 역일(calendar day)로 2일을 초과하여 연속적으로 인공호흡기를 적용 받은 만 15세 이상의 환자이다[19,20]. 단기간 수술 후 안정화 또는 일시적 모니터링을 목적으로 운영되는 뇌졸중집중치료실과 응급중환자실은 연구 대상에서 제외하여 지속적인 중환자 치료를 제공하는 중환자실만을 포함하였다. 또한 체외막산소공급(extracorporeal membrane oxygenation, ECMO) 적용 환자는 CDC/NHSN VAE 감시 제외기준에 따라 본 연구에서도 제외하였다. 이는 ECMO 적용 시 FiO₂와 PEEP의 변화가 환자의 실제 폐 상태 변화를 정확히 반영하지 않을 수 있기 때문이다[5]. 또한 비침습적 인공호흡기(intermittent positive-pressure breathing, nasal positive end-expiratory pressure, continuous positive airway pressure 등)만을 적용한 환자, 그리고 진단기준에 해당하는 징후 또는 증상이 중환자실 입실 전이나 입실 후 2일 이내 발생한 경우도 제외하였다[6,19,20].

### 3. 감시 정의

#### 1) 사례 정의

**Figure 1.**
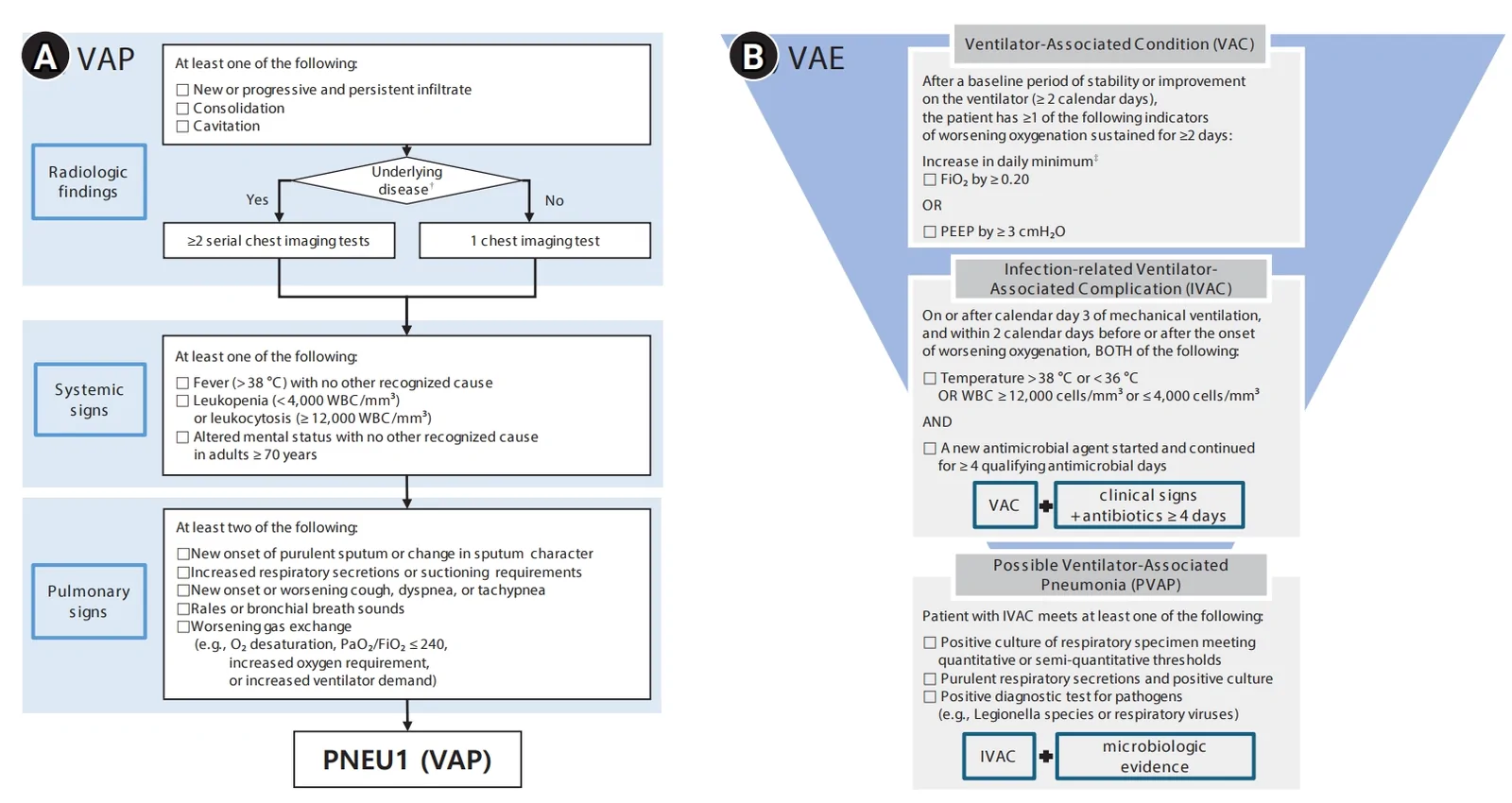
Surveillance criteria for (A) ventilator-associated pneumonia (VAP) and (B) ventilator-associated events (VAE) according to Centers for Disease Control and Prevention/National Healthcare Safety Network criteria. Panel A shows the surveillance criteria for VAP, including radiologic, systemic, and pulmonary findings. Panel B shows the hierarchical structure of VAE, including ventilator-associated condition (VAC), infection-related ventilator-associated complication (IVAC), and possible ventilator-associated pneumonia (PVAP). PNEU1 = Clinically defined pneumonia; WBC = White blood cell. ^†^Underlined diseases indicate underlying cardiopulmonary diseases; ^‡^Daily minimum is defined as the lowest value of FiO₂ or PEEP during a calendar day that is maintained for >1 hour.

VAP는 CDC/NHSN과 KONIS에서 제시한 ‘임상적으로 정의된 폐렴(PNEU1)’ 진단기준을 따라 정의하였다[19,20]. VAE는 CDC/NHSN에서 제시한 감시 정의를 따랐다[6]. VAE 감시체계는 VAC, IVAC, PVAP을 위계적으로 모두 감시하였다(Figure 1).

본 연구에서는 VAE 발생 분석을 위해서 기존 VAE 감시 연구에서 활용한 방법에 따라, ① VAC-Plus (IVAC 및 PVAP을 포함한 모든 VAC 사건), ② IVAC-Plus (PVAP을 포함한 모든 IVAC 사건), ③ PVAP (PVAP에 해당하는 사건) 세 개의 위계적 범주로 정의하였다[6,15]. 또한 CDC/NHSN과 KONIS의 진단기준에 따라 VAP와 VAE 모두 동일 환자에서 14일 이내 반복적으로 발생한 사건은 반복감염기간(repeat infection timeframe) 개념을 적용하여 하나의 사건으로 정의하였다[6,19,20].

#### 2) 인공호흡기 사용일수 정의

VAP와 VAE 발생률은 인공호흡기 사용일수 기준으로 산출하였다. 인공호흡기 사용일수는 KONIS 기준에 따라 기관내 삽관 또는 기관절개를 통해 인공호흡기를 적용 받은 환자를 대상으로, 매일 00시를 기준으로 의무기록을 확인하여 산출하였다. 각 환자의 인공호흡기 사용기간을 역일(calendar day) 기준으로 계산하여 합산한 값을 총 인공호흡기 사용일수로 정의하였다. 인공호흡기가 일시적으로 중단되었더라도 역일(calendar day) 기준 1일 이내에 다시 적용된 경우에는 연속적으로 인공호흡기를 적용한 것으로 간주하였다[19,20].

#### 3) 감염 발생률 정의

CDC/NHSN과 KONIS 감염 발생률 산출 공식에 따라. VAP 발생률은 1,000 인공호흡기 사용일수당 발생건수로 정의하였다(VAP발생률 = VAP발생건수/총 인공호흡기 사용일수×1,000)[19,20]. VAE 발생률은 1,000 인공호흡기 사용일수당 VAE 발생건수로 정의하였다(VAE 발생률 = VAE발생건수/총 인공호흡기 사용일수×1,000). VAE는 진단 범주에 따라 VAC-Plus, IVAC-Plus 및 PVAP으로 구분하였으며, 각 발생률은 동일한 방식으로 산출하였다[6].

### 4. 연구변수와 도구

#### 1) 대상자 특성 및 치료 관련 변수

대상자의 일반적 특성으로는 연령, 성별, 동반질환(고혈압, 뇌혈관질환), 진료과(내과, 외과, 기타), 중환자실 유형(중환자실, 신경계 중환자실, 심혈관계 중환자실, 심뇌혈관계 중환자실) 및 질병 중증도를 포함하였다. 연령은 만 나이를 기준으로 조사하였으며, 세계보건기구(World Health Organization)와 경제협력개발기구(Organisation for Economic Co-operation and Development)의 기준[21,22]의 연령군을 참고하여 청년(≤ 39세), 중장년(40~64세), 노년(≥ 65세)의 세 군으로 범주화하였다. 질병 중증도는 중환자 중증도 평가 도구인 Acute Physiology and Chronic Health Evaluation II (APACHE II)로 측정한 총점(범위 0~71점)으로 평가하였다.

치료 관련 변수로는 인공호흡기 사용기간, 조기 경장영양 및 스테로이드 사용 여부, 중환자실 재원기간을 포함하였다. 인공호흡기 사용기간은 중환자실 재원기간 중 적용된 각 환자의 총 인공호흡기 사용일수로 정의하고 연속형 변수로 수집하였다. 또한 기존 연구와의 비교를 위해 선행연구 및 자료의 분포를 고려하여 6개의 구간(3~7일, 8~14일, 15~21일, 22~28일, 29~56일, ≥ 57일)으로 범주화하였다[13,15]. 조기 경장영양은 유럽임상영양대사학회(European Society for Clinical Nutrition and Metabolism) 가이드라인에 따라 중환자실 입실 후 48시간 이내에 경장영양을 시작한 경우로 정의하였다[23].

#### 2) VAE와 VAP의 감시 관련 변수

VAP 감시 관련 변수는 흉부 영상 소견, 발열, 백혈구 수치 변화 및 호흡기 분비물 증가 여부를 포함하였다[19,20]. 흉부 영상 소견과 백혈구 수치 변화는 검사결과기록지를, 발열은 임상관찰기록지를, 호흡기 분비물 증가 여부는 간호기록지를 이용하여 확인하였다.

VAE 감시 관련 변수는 최소 FiO₂, 최소 PEEP, 체온, 백혈구 수치, 신규 항생제 사용 여부, 객담 배양검사 결과, 호흡기 바이러스 everse transcription polymerase chain reaction (RT-PCR) 검사 결과 및 소변 레지오넬라 검사 결과를 포함하였다[6]. 최소 FiO₂, 최소 PEEP 및 체온은 임상관찰기록지를, 백혈구 수치와 객담 배양검사, 호흡기 바이러스 RT-PCR 및 소변 레지오넬라 검사 결과는 검사결과기록지를, 신규 항생제 사용 여부는 처방기록지를 이용하여 확인하였다.

#### 3) 깊은 진정

깊은 진정은 중환자의 의식수준 및 진정-초조 정도를 측정하는 11점 척도인 Richmond Agitation–Sedation Scale (RASS)로 평가하였으며 RASS는 점수가 높을수록 초조 상태를, 낮을수록 진정 상태를 의미하고, 일반적으로 +1점 이상은 초조, 0점은 안정, −1점 이하는 진정 상태를 나타낸다[24,25]. 본 연구에서는 측정한 값이 -3점 이하인 경우를 깊은 진정으로 정의하였다(RASS ≤ -3). RASS평가는 중환자실 재원기간 동안 8시간 간격(하루 3회)으로 중환자실 간호사에 의해 수행되어 기록되었으며, 본 연구에서는 해당 의무기록에서의 값을 수집하였다.

깊은 진정 비율은 중환자실 재원기간 동안 깊은 진정의 노출 정도를 평가하기 위해 산출하였으며, 재원기간 전체에 걸쳐 기록된 RASS 평가를 이용하였다. 구체적으로, 중환자실 재원기간 동안 깊은 진정에 해당한 RASS 평가 횟수를 전체 RASS 평가 횟수로 나누어 계산하였다(깊은 진정 비율 = 깊은 진정 평가 횟수/전체 RASS 평가 횟수×100). 또한 깊은 진정 노출 시점을 구분하기 위해 중환자실 입실 후 초기 3일 및 7일 동안의 깊은 진정 비율도 각각 동일한 방식으로 산출하였다.

깊은 진정일 비율은 깊은 진정의 누적 노출을 반영하기 위해 산출하였다. 하루 동안 기록된 RASS 평가 중 절반 이상이 깊은 진정(RASS ≤ -3)에 해당하는 경우 해당 날짜를 깊은 진정일로 정의하였으며, 전체 중환자실 재원기간 대비 비율로 계산하였다(깊은 진정일 비율 = 깊은 진정으로 평가된 일 수/전체 중환자실 입원일 수×100).

#### 4) 감시시간

감시시간은 연구자가 VAP와 VAE 감시를 수행하는 데 소요된 시간으로 정의하였다. 감시시간 측정의 일관성을 유지하기 위해 VAP와 VAE 감시는 동일 연구자가 수행하였다. 감시시간 측정에 대한 표준화된 평가기준이 없어, 본 연구에서는 Klompas 등[8]의 연구에서 사용한 감시시간 평가 방법을 참고하여 측정하였다. 타이머를 이용하여 전체 대상 환자에 대한 VAP 및 VAE 감시에 소요된 총 시간을 분 단위로 기록하였다. VAP 및 VAE 감시시간은 감시대상 환자 수로 나누어 환자당 평균 감시시간으로 산출하였다(VAP 평균 감시시간 = 총 VAP 감시시간/감시대상 환자 수; VAE 평균 감시시간 = 총 VAE 감시시간/감시대상 환자 수). 단, VAP 감시 과정에서 감염 여부 판단이 모호하여 연구자와 감염관리 전문간호사 간 추가 사례 검토가 이루어진 경우 해당 검토에 소요된 시간은 총 감시시간 산출에서 제외하였다. 이는 VAP 감시의 추가 사례 검토로 측정 비뚤림은 줄이되, 감시시간은 보수적으로 추정하기 위함이었다.

### 5. 자료 수집

본 연구에서는 감염감시 및 대상자 특성 분석을 위한 연구변수를 의무기록을 이용하여 후향적으로 수집하였다. 자료는 연구대상자 선정 기준에 따라 응급중환자실 및 뇌졸중집중치료실 입실 환자와 비침습적 인공호흡기 적용 환자를 제외한 중환자실 입실 환자의 자료를 의무기록실로부터 제공받았다. 자료 수집은 동아대학교병원 기관생명윤리위원회의 승인을 받은 후 수행되었다.

제공받은 자료를 바탕으로 연구자가 독립적으로 감시를 수행하였으며 감시는 2025년 4월 1일부터 2025년 6월 30일 동안 수행하였다. 감시의 신뢰성과 일관성을 위해 연구자는 감염관리 전문가의 감독 하에 연구 전 VAP와 VAE 감시 진단관련 표준화된 교육을 받았다. VAP는 KONIS 기준에 따라 발생 여부를 판정하고, KONIS 감염환자기록지에 기록하였다[19,20]. VAP 사례 중 감염 여부 판단이 모호한 경우에는 연구자와 감염관리실 근무 및 중환자실 감염감시 경력이 있는 전문간호사가 함께 의무기록을 재검토하여 최종 진단하였다. VAE는 CDC/NHSN 기준에 따라 감시하였으며, CDC/NHSN에서 제공하는 VAE calculator (version 12.0)를 이용하여 발생 여부를 판정한 후 VAE 증례기록지에 기록하였다[6,26]. 감시시간은 타이머를 이용하여 VAP 및 VAE 감시 시작부터 종료까지의 시간을 측정하고, 이를 누적하여 총 감시시간을 산출하였다. 동일 대상자에서 VAP와 VAE를 모두 감시하므로 순서에 따른 편향을 최소화하기 위해 감시기간을 2개의 기간으로 나누어 수행하였다. 상반기(1월~6월)에는 VAP 감시를 먼저 수행한 후 VAE를 감시하였고, 하반기(7월~12월)에는 순서를 반대로 수행하였다.

### 6. 자료 분석

인공호흡기 사용일수를 산출한 후, 이를 분모로 하여 VAP 및 VAE의 발생률(인공호흡기 1,000일당 발생건수)을 산출하여 비교하였다. 또한 VAP 감시 결과를 기준으로 VAC-Plus의 민감도와 양성예측도를 산출하였으며, VAP와 VAE 감시에 소요되는 평균 감시시간(분)을 비교하였다. 발생률 분석은 사건(event episodes)을 분석 단위로 수행하였으며, VAP 및 VAE와 관련된 요인 분석은 환자를 분석 단위로 수행하여 동일 환자에서 발생한 반복 사건은 제외하고 첫 번째 사건만 포함하였다.

대상자의 일반적 특성 및 치료관련 변수는 빈도와 백분율, 중앙값과 사분위수의 기술통계를 제시하였다. 또한 VAP와 VAE 발생 여부에 따른 일반적 특성과 치료관련 변수는 기술통계를 제시한 후 집단 간 비교는 Mann–Whitney U 검정, 카이 제곱 검정 및 Fisher의 정확 검정 또는 Monte Carlo방법을 이용하였다.

VAP 및 VAE와 관련된 요인을 확인하기 위해 다변량 이항 로지스틱 회귀분석을 수행하였다. 각 회귀모형에는 선행연구와 임상적 타당성을 고려하여 선정한 주요 연구변수와, 단변량 분석 및 문헌고찰을 통해 잠재적 교란변수로 판단된 성별, 연령군, 동반질환 및 진료과를 포함하였다. 분석 결과는 교정된 오즈비(adjusted odds ratio)와 95% 신뢰구간(confidence interval)으로 제시하였다. 모든 통계 분석은 양측 검정으로 수행하였으며, 통계적 유의수준은 *p* < .050로 설정하였다. 통계 분석은 IBM SPSS Statistics version 29.0 (IBM Corp., Armonk, NY, USA)을 이용하였다.

### 7. 윤리적 고려

본 연구는 동아대학교병원 기관생명윤리위원회의 승인을 받은 후 수행되었다(IRB No. DAUHIRB-25-015). 본 연구는 후향적 연구로 EHR 자료를 이용하여 수행되었으므로 대상자 동의는 면제되었다. 자료 수집 과정에서는 환자 등록번호를 포함한 EHR 자료를 이용하였으며, 이름, 주민등록번호 등 개인식별정보는 포함되지 않았다. 수집된 자료는 연구 목적에 한하여 사용되었고, 비밀번호로 보호된 파일 형태로 저장하여 연구자 외에는 접근할 수 없도록 제한하였다. 연구 종료 후 일정 기간 보관한 뒤 모든 전자자료는 영구 삭제하고 문서 자료는 파쇄하여 폐기할 예정이다.

## 연구 결과

### 1. 연구 대상자 및 감시 소요 시간

연구 기간 동안 중환자실 입실 2,219명 중 1,003명의 환자가 역일(calendar day) 2일 초과 연속적으로 인공호흡기를 적용 받았다. 이 중 ECMO를 적용 받은 61명을 제외한 942명이 최종 분석 대상에 포함되었다.

연구 기간 동안 VAP 31건과 VAE 84건이 확인되었다. 감시시간을 비교한 결과 총 감시시간은 VAP 2,401분, VAE 613분으로 VAP 감시에 더 많은 시간이 소요되었다. 환자당 평균 감시시간은 VAP 2.5분, VAE 0.7분이었다.

### 2. VAP 및 VAE 발생률

**Figure 2.**
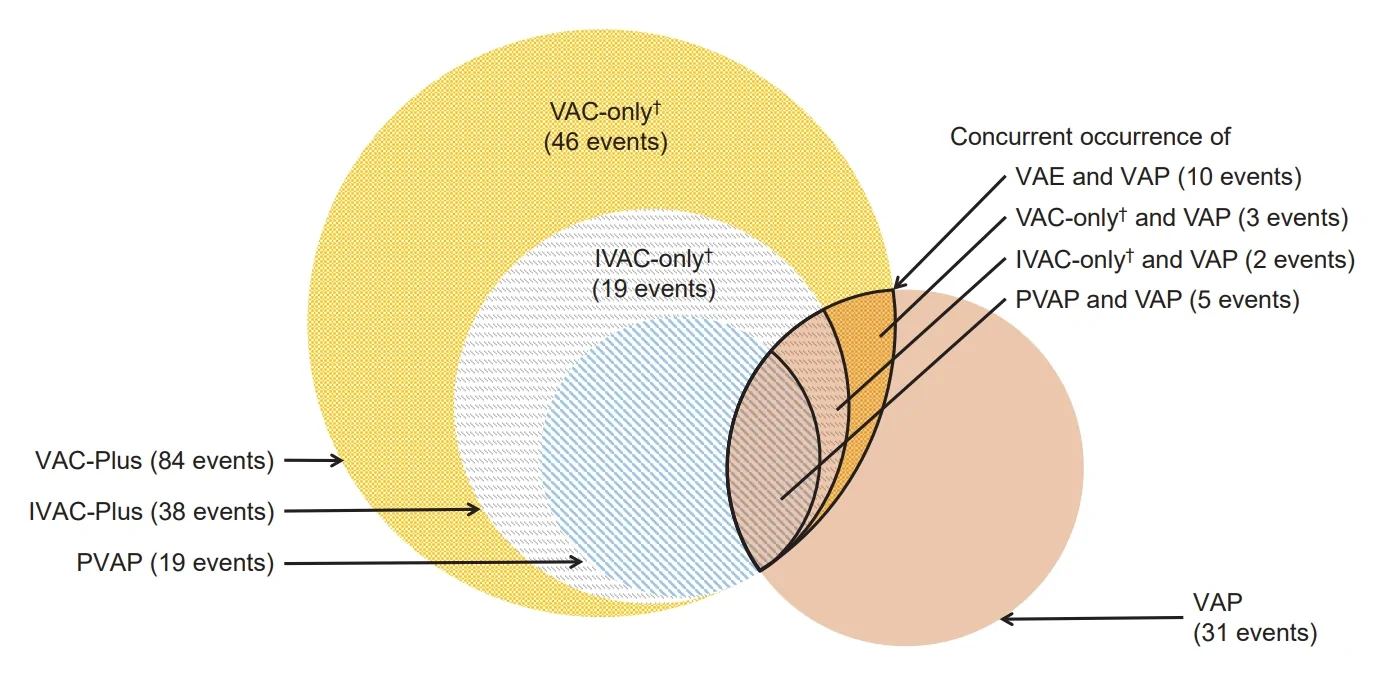
Distribution and overlap of ventilator-associated event (VAE) and ventilator-associated pneumonia (VAP) event episodes. A total of 84 VAE episodes were identified, including 46 VAC-only, 19 IVAC-only, and 19 PVAP episodes. Thirty-one VAP episodes were detected, of which 10 occurred concurrently with VAE. VAC = Ventilator-associated condition; IVAC = Infection-related ventilator-associated complication; PVAP = Possible ventilator-associated pneumonia. ^†^Only indicates events that met the criteria for a given category but did not meet the criteria for higher-tier categories.

총 10,542 인공호흡기 사용일수 동안 VAP 발생률은 1,000 인공호흡기일수당 2.9건, VAE 발생률은 8.0건으로 나타났다. VAP는 31건이었으며, VAE는 VAC-Plus 84건, IVAC-Plus 38건, PVAP 19건으로 확인되었다. VAP와 VAE가 동시에 발생한 사건은 10건이었다. VAP를 기준으로 하였을 때 VAC-Plus의 민감도는 32.3%(10/31건), 양성예측도는 11.9%(10/84건)였다(Figure 2).

중환자실 유형에 따른 발생률을 비교한 결과 VAP 발생률은 신경계 중환자실에서 가장 높았으며(1,000 인공호흡기일수당 5.2), VAE 발생률은 심혈관계 중환자실에서 가장 높았다(1,000 인공호흡기일수당 9.7). 또한 모든 중환자실에서 인공호흡기 사용기간에 따른 발생률은 지표별로 서로 다른 양상을 보였다. VAP 발생률은 15~21일 구간에서 가장 높았으며 이후 감소하는 양상을 보였다. VAC-Plus 발생률은 8~14일 구간에서 가장 높았으며, 이후에는 뚜렷한 증감 경향 없이 구간별로 다소 변동하는 양상을 보였다. IVAC-Plus 발생률은 15~28일 구간에서 비교적 높은 수준을 유지한 후 감소하였으며, PVAP 발생률은 22~28일 구간에서 가장 높았다(Table 1).

### 3. VAP 및 VAE 관련 요인

단변량 분석에서 VAP 발생은 성별, 연령군, 동반질환, 진료과, 인공호흡기 사용기간, 스테로이드 사용, 입실 후 3일 및 7일의 깊은 진정 비율, 그리고 중환자실 재원기간과 유의한 관련을 보였다. VAE의 경우 동반질환, 진료과, 인공호흡기 사용기간, 스테로이드 사용, 깊은 진정 비율(입실 후 3일 및 7일 포함) 및 깊은 진정일 비율 그리고 중환자실 재원기간과 유의한 관련을 보였다(Table 2).

다변량 로지스틱 회귀분석에서는 주요 연구변수와 함께 교란변수를 보정하여 분석하였다. 주요 연구변수로는 스테로이드 사용, 인공호흡기 사용기간, 깊은 진정일 비율을 포함하였다. 교란변수로는 성별, 연령군, 동반질환(고혈압, 심뇌혈관질환), 진료과를 포함하였다. 인공호흡기 사용기간과 중환자실 재원기간 사이에 높은 상관관계(ρ = .767, *p* < .001)가 확인되어 다중공선성을 최소화하기 위해 최종 모형에는 인공호흡기 사용기간만을 포함하였다.

다변량 분석 결과 인공호흡기 사용기간은 VAP와 VAE 모두에서 유의한 관련성을 보였다. 반면 깊은 진정일 비율은 VAE 발생과 관련이 있었으며, 하위 범주인 VAC-Plus, IVAC-Plus 및 PVAP 발생과도 일관된 관련성을 보였다. 또한 스테로이드 사용은 VAP 및 PVAP 발생과 관련이 있었다.

모형의 설명력은 Nagelkerke R² 기준으로 VAP모형이 .18이었으며, VAE 범주에서는 VAC-Plus .20, IVAC-Plus .24, PVAP .27로 나타났다. Hosmer-Lemeshow 검정 결과 모든 모형은 자료에 적합한 것으로 확인되었다(*p* > .050) (Table 3).

## 논의

본 연구는 단일 상급종합병원의 4개 성인 중환자실을 대상으로 VAP와 VAE의 발생률, 관련 요인 및 감시시간을 비교하여 두 감시체계의 특성을 분석하였다. 연구 결과 VAE는 VAP보다 약 세 배 더 빈번하게 발생하였음에도 불구하고, 감시시간은 VAP에 비해 약 4분의 1 수준으로 짧았다. 또한 관련 요인 분석 결과, VAP와 VAE의 감시체계에서 일부 공통된 관련 요인을 공유하였으나, 임상적 필요로 조절이 제한되는 스테로이드 사용은 VAP 및 PVAP 발생과 관련된 반면, 중재를 통해 조절 가능한 깊은 진정일 비율은 VAE 발생과 관련이 있었다.

본 연구에서 확인된 VAP 발생률은 1,000 인공호흡기 사용일수당 2.9건으로 나타났다. 국외 체계적 문헌고찰 연구[27]에서 VAP 발생률을 1,000 인공호흡기 사용일수당 7~43건으로 보고하였다. 아시아 지역을 대상으로 한 다기관 메타분석[28]에서도 전체 VAP 발생률은 1,000 인공호흡기 사용일수당 15.1건, 고소득 국가만을 포함한 경우에도 9.0건으로 보고하였다. 따라서 본 연구 결과는 이러한 선행연구들과 비교할 때 상대적으로 낮은 수준이었다. 반면 본 연구에서의 VAP 발생률은 국가 의료관련감염 감시체계인 KONIS와 CDC/NHSN 자료에서 보고된 수치와 비교했을 때 상당히 높은 수준이었다. 국내 KONIS 감시연보에서는 900병상 이상 의료기관에서 1,000 인공호흡기 사용일수당 0.95건, 미국 CDC/NHSN 자료에서는 교육병원 중환자실에서 1,000 인공호흡기 사용일수당 1.6건으로 보고되었다[29,30]. 또한 국내에서는 900병상 이상 의료기관의 50% 이상에서, 미국에서는 비교육병원의 절반 이상에서 VAP 발생이 0건으로 보고된 바 있다. 이러한 국가 의료관련감염 감시체계의 낮은 보고 수준은 진단기준의 엄격한 적용과 보고과정에서의 과소보고의 가능성과 관련될 수 있음이 기존 연구에서도 지적되어왔다[5,8]. 이에 KONIS에서는 국가 의료관련감염 감시체계에서의 과소보고 문제를 보완하기 위해 격년으로 내•외부 타당도 조사를 시행하고 있으나[4], 감염관리실 설치와 인력배치 기준 확대에 따른 잦은 감시 인력 교체와 신규 인력의 대거 유입으로 인해 여전히 VAP의 민감도는 낮은 수준으로 보고되고 있다. 따라서 감시데이터의 정확성 유지를 위해 지속적인 감시자 교육이 필요할 수 있다.

본 연구에서 확인된 VAE 발생률은 1,000 인공호흡기 사용일수당 8.0건으로 나타났다. 이는 유럽연합에서 수행된 EUVAE study [17]에서 보고된 VAE 발생률(1,000 인공호흡기 사용일수당 40.9건)과 He 등[15]의 아시아 대규모 연구에서 보고한 VAE 발생률(16.7건)에 비해 낮은 수준이었으며, 2014년 CDC/NHSN에서 보고된 교육병원 내외과 중환자실의 평균 VAE 발생률(7.8건)과는 유사한 수준이었다[31]. 또한 CDC/NHSN의 보고에 따르면 일부 기관(약 25%)에서는 VAE 발생이 0건으로 보고되었으나 대다수(75% 이상)에서는 발생이 보고되었고, 15병상 이하의 소규모 중환자실에서조차도 절반 이상에서 VAE 발생이 확인되었다[31]. 이는 VAE 감시가 소규모 의료기관을 포함한 대부분의 의료기관에서 발생이 보고된다는 점에서 인공호흡기 관련 상태 변화를 폭넓게 포착하며, 다양한 중환자실 환경에서 활용 가능한 일관성 있는 감시지표임을 시사한다.

본 연구에서는 VAP와 VAE 모두 가을철에 발생률이 높게 나타났다. 이는 연구 기간 동안 coronavirus disease 2019 (COVID-19) 유행에 따른 중환자실 진료 환경의 변화가 일부 반영된 결과일 수 있으며, 본 연구에서 확인된 스테로이드 사용 및 인공호흡기 사용기간과 VAP 또는 VAE 간의 관련성은 해석에 주의가 필요하다[32–34].

본 연구에서 VAP의 경우 신경계 중환자실에서 1,000 인공호흡기 사용일수당 5.2건으로 높은 발생률을 보였다. 이는 의식 저하와 기도 보호 능력 감소로 인한 흡인 위험의 증가, 장기간 인공호흡기 사용과 같은 환자의 특성과 관련될 수 있다[1,15]. 반면 VAE는 심혈관계 중환자실에서 1,000 인공호흡기 사용일수당 9.7건으로 높은 발생률을 보였다. 이는 수혈 및 수액과다와 같은 치료 과정 요인과 이에 따른 폐부종 등의 변화로 설명될 수 있다[13,35]. 본 연구의 이러한 결과는 대상 환자의 특성과 치료 과정을 반영한 인공호흡기 관련 합병증 예방 및 감시 전략이 필요할 수 있음을 시사한다.

본 연구에서 VAC-Plus 기준의 VAE 감시는 VAP을 참조 기준으로 평가했을 때 민감도와 양성예측도가 모두 낮아, 두 감시체계 간 일치도가 제한적인 것으로 나타났다. 이러한 결과는 VAE와 VAP 감시체계 간 일치도가 낮다고 보고한 기존 연구들과 일관된 경향을 보인다. Fan 등[11]의 체계적 문헌고찰 및 메타분석에서는 VAP 진단에 대한 VAC와 IVAC의 민감도가 각각 41.8%와 36.3%였으며, 양성예측도는 23.2%로 낮게 보고되었다. 국내 Lee [10]의 연구에서도 VAE의 민감도는 약 38%, 양성예측도는 7%에 불과하여 매우 낮은 수준을 보였다. 또한 유럽 다기관 연구[17]에서는 VAE가 실제 VAP 환자의 약 3명 중 1명만을 포착하는 것으로 나타났으며, Weinberger 등[13]의 문헌고찰에서도 VAE 환자 중 실제 폐렴이 원인인 비율이 21%에서 62% 수준에 그치는 것으로 확인되었다. 이러한 결과는 두 감시체계가 동일한 임상 사건을 반영하지 않음을 시사한다. 다만, 본 연구에서 제시한 민감도와 양성예측도는 VAP를 기준으로 새롭게 도입된 VAE 감시체계가 어느 정도 중첩되는지를 평가하기 위한 참고지표로 산출한 것이므로 VAP에 대한 VAE의 진단 정확도를 평가한 것으로 해석하기보다는 두 감시체계 간의 관계를 이해하기 위한 참고자료로 이해하는 것이 바람직할 것이다.

VAE 감시는 감염 여부와 관계없이 인공호흡기 사용 중 발생하는 다양한 임상적 악화를 포착하기 위한 감시 체계이며[13,36], VAC, IVAC, PVAP으로 이어지는 위계적 구조를 통해 상태 변화를 단계적으로 구분하여 평가할 수 있다[5,6,8]. 이러한 감시체계 구조는 인공호흡기 치료 과정에서 발생하는 환자 상태 악화의 연속적인 궤적(trajectory)을 따라감으로써 폐렴 발생 이전 단계에서 조기 중재로 이어질 수 있게 하며 환자 안전 측면에서 중요한 의미를 가진다[36,37]. 반면 VAP 감시는 환자의 임상적 폐렴 진단과 경험적 항생제 치료의 시작 시점을 결정하는데 밀접하게 연관된 지표로서의 의미를 가진다[3,14]. 특히 중환자실에서 VAP를 포함한 호흡기 감염 의심 사례는 광범위 항생제 사용의 주요 원인이 되므로, 정확한 VAP 진단과 평가는 환자의 예후 개선뿐만 아니라 불필요한 항생제 사용을 줄이고 다제내성균 발생을 억제하는 항생제 관리 측면에서 여전히 중요한 임상적 가치를 지닌다[1,3]. 이에 따라 중환자실 폐렴 감시에서는 감시 목적에 따라 두 지표를 상호보완적으로 활용하는 것이 바람직하다.

본 연구에서 VAP 감시에 비해 VAE 감시시간이 약 4분의 1 수준으로 짧았다. 기존 연구에서도 VAE 감시는 VAP에 비해 감시시간이 짧아서, 국내 Lee [10]의 연구에서 감시시간은 VAP 39분 대비 VAE 10.3분, 미국 Klompas 등의 연구[8]에서도 VAP 39분 대비 VAC 1.8분으로 나타나 본 연구 결과와 일관된 경향을 보였다. 그러나 본 연구에서 측정된 VAP 및 VAE의 평균 감시시간(VAP 2.5분, VAE 0.7분)은 모두 선행연구에 비해 현저히 짧았다. 본 연구에서 연구기관의 중환자실 EHR이 고도화되어 있었고, CDC/NHSN VAE calculator 사용으로 감시시간을 단축할 수 있었던 것과 관련이 있을 수 있다. 반면 Klompas 등[8]의 연구는 수기 감시를 기반으로 수행되었고, Lee [10]의 연구 또한 VAE calculator와 수기 감시를 병행하였다. 그럼에도 VAE 감시시간은 VAP의 약 4분의 1 수준이었는데, 이는 VAE가 객관적인 인공호흡기 설정값을 기반으로 감시되는 반면 VAP는 영상 소견, 임상 증상 및 의무기록 검토가 필요하기 때문으로 판단된다. 국내 연구에서는 환자 상태에 따라 FiO₂와 PEEP이 수시로 변화하기 때문에 이를 의무기록에서 확인하기 어렵고, 전산화 수준이 낮은 의료기관에서는 VAE 적용이 어려울 수 있다는 지적이 있었다[38]. 그러나 2020년 한국보건의료정보원의 보건의료정보화 실태조사에 따르면 상급종합병원 100%, 종합병원 96% 수준으로 EHR 도입이 이루어져[39], VAE 감시체계를 전산화 및 자동화하여 적용할 수 있는 기반이 상당 부분 마련되어 있는 것으로 볼 수 있다. 이러한 환경에서는 VAE 감시가 더 적은 시간으로 수행될 수 있으며, EHR 기반 자동화를 통해 감시 정확성을 향상시킬 수 있을 것으로 기대된다.

감시체계의 우수성을 평가할 때는 정확성, 효율성뿐 아니라 임상적 활용 가능성도 함께 고려할 필요가 있다. 최근 감염 감시체계는 객관성과 재현성을 높이고 전산화 및 자동화를 통해 감시 효율성을 향상시키는 방향으로 발전하고 있다[5,31]. 미국의료역학회(Society for Healthcare Epidemiology of America, SHEA), 미국감염학회(Infectious Diseases Society of America, IDSA), 미국감염관리·역학전문가협회(Association for Professionals in Infection Control and Epidemiology, APIC)가 공동으로 발표한 2022년 가이드라인에는 VAE 감시체계가 객관적이고 재현 가능하며 자동화가 가능하도록 설계되었음을 강조하고, EHR 기반 자동화 감시를 권고하는 동시에 제한된 감염관리 자원을 효율적으로 활용하여 환자 결과를 개선할 수 있는 감시체계의 중요성을 제시하고 있다[40]. 이러한 측면에서 VAE 감시는 감시 수행에 필요한 시간과 자원을 절감하면서도 정확한 감시를 가능하게 하는 효율적인 감시체계로 활용될 수 있을 것으로 사료된다.

본 연구에서는 인공호흡기 사용기간이 VAP와 VAE 모두에서 공통적인 관련 요인으로 확인되었다. 이는 인공호흡기 사용기간이 길어질수록 인공호흡기 관련 합병증 발생 위험이 증가한다는 기존 연구 결과와 일치한다[12,13,17,41]. 따라서 불필요한 인공호흡기 사용을 최소화하고 조기 이탈을 촉진하기 위한 전략이 필요할 수 있다.

본 연구에서는 스테로이드 사용이 VAP와 PVAP 발생의 관련 요인으로 나타났다. 다만 본 연구는 COVID-19 유행 시기에 수행되어 스테로이드 사용이 직접적인 관련 요인이라기보다 환자의 질병 중증도를 반영했을 가능성도 있다[32–34]. 그럼에도 스테로이드 사용과 감염 관련 사건 간의 연관성은 감시 자료 해석과 예방 전략 수립 시 치료 관련 요인을 함께 고려해야 함을 시사한다. 특히 스테로이드 사용이 증가하는 상황에서는 VAP 예방 전략과 감시가 더욱 중요할 수 있다.

본 연구에서는 중환자실 재원기간 동안의 깊은 진정일 비율이 VAC-Plus, IVAC-Plus 및 PVAP과 유의한 관련성을 보였다. 또한 깊은 진정 관련 모든 변수들을 이용한 민감도 분석에서도 깊은 진정과 VAE 간의 관련성은 일관되게 나타났다. 기존 연구에서는 주로 삽관 직후 기간에 초점을 두어 삽관 후 48시간 이내의 깊은 진정이 재원 기간 연장이나 사망률 증가와 같은 부정적인 임상 결과와 관련된다고 보고되었다[24,25]. 본 연구 결과는 이러한 선행 연구 결과에서 더 확장되어, 깊은 진정과 VAE 간의 관련성이 초기 48시간에 국한되지 않고 인공호흡기 사용기간 전반에 걸쳐 관찰될 가능성을 시사한다. 깊은 진정은 측정 가능하고, 중재를 통해 조절할 수 있는 요인이라는 점에서 임상적으로 중요하다. 최근 진정 관리 지침에서는 가능한 얕은 진정을 유지하고 매일 진정 수준을 재평가하여 최소화할 것을 권고하고 있다[42–45]. 이러한 권고를 고려할 때, EHR을 통해 진정 상태가 일상적으로 기록되는 환경에서는 삽관 직후뿐 아니라 인공호흡기 사용기간 동안 진정 수준을 지속적으로 평가하고, 가능한 경우 깊은 진정을 최소화하려는 관리 전략을 고려할 필요가 있다.

VAP와 VAE는 서로 다른 감시체계로, 이를 상호보완적으로 활용하는 것은 감염 진단 중심의 감시와 인공호흡기 관련 상태 악화 감시를 함께 반영한다는 점에서 중요하다. 임상적으로 정의된 폐렴(PNEU1) 진단기준을 적용한 VAP는 감염의 진단과 항생제 치료 결정, 기관 간 비교에 필수적인 지표이다[14]. 반면 VAE 감시체계는 VAC, IVAC, PVAP으로 이어지는 위계적 구조를 통해 감염성 및 비감염성 합병증을 모두 포착할 수 있어 보다 넓은 환자 안전 문제를 반영하며, 감시 부담이 적은 지속적 모니터링과 조기 인지에 적합하다[36,37,46]. 또한 VAE 감시는 수정 가능한 관련 요인을 보다 조기에 확인할 수 있는 예방 중심 감시 접근법으로 활용될 수 있다[46]. 이러한 특성은 진정 관리, 자발호흡 평가, 체위 유지, 구강간호 등 인공호흡기 관리 번들 중재와 연계되어 선제적 개입을 가능하게 한다. 실제로 인공호흡기 관리 번들을 적용한 연구에서 IVAC 및 PVAP 발생이 감소한 것으로 보고되어, 이러한 접근의 임상적 유용성을 뒷받침한다[47]. 특히 VAE는 인공호흡기 설정값과 검사 결과 등 EHR에서 확인 가능한 자료를 기반으로 하므로, EHR 기반 감시 환경에서 FiO₂나 PEEP 변화와 같은 지표를 자동으로 탐지하는 데 활용될 수 있다. 이를 통해 VAC 발생 시 환자 상태 악화를 보다 신속하게 인지하고, 진정 수준이나 인공호흡기 설정 재평가 등의 임상적 대응에 활용될 수 있다.

본 연구에는 몇 가지 제한점이 있다. 첫째, 단일 기관에서 수행된 연구이며 관찰 기간이 1년으로 비교적 짧아 연구 결과의 일반화에는 제한이 있을 수 있다. 또한 본 연구는 EHR 기반 감시체계가 잘 구축된 상급종합병원 환경에서 수행되었으므로, 전산화 수준이 낮거나 감시자료 접근이 제한적인 의료기관에 동일하게 적용하기에는 한계가 있다. 둘째, 연구가 COVID-19 유행 시기에 수행되어 환자 중증도, 인공호흡기 사용 양상, 스테로이드 사용 등이 영향을 받았을 가능성이 있다. 따라서 결과 해석에 주의가 필요하며, 반복연구가 필요하다. 셋째, 본 연구는 후향적 관찰연구로 일부 변수(인공호흡기 사용기간, 깊은 진정 비율, 중환자실 재원기간 등)에 사건 발생 이후의 정보가 포함되어 있어 시간의존성 비뚤림(time-dependent bias)과 역인과성(reverse causality)을 완전히 배제하기 어렵다. 다만 본 연구에서는 인공호흡기 사용일수로 보정하여 발생률을 산출하고자 하였으나, 후향적 연구 특성상 변수와 결과 간 인과관계를 명확히 규명하는 데 한계가 있다. 향후 전향적 연구 또는 시간의존성을 고려한 분석이 필요하다. 넷째, 본 연구의 다변량 회귀분석에서 VAC-Plus를 제외한 결과는 사건 수 대비 변수 수(events per variable)가 권고 수준에 미치지 못하여 추정치의 불안정성과 과적합(overfitting) 가능성을 완전히 배제하기 어렵다[48]. 따라서 관련 요인에 대한 결과 해석에는 주의가 필요하다. 향후 다기관 및 다년간 연구를 통해 관련 요인의 재현성을 검증할 필요가 있다. 그럼에도 표준화된 감시 프로토콜을 적용하고 다양한 중환자실을 포함한 동일 코호트에서 두 감시체계를 직접 비교하였다는 점은 본 연구의 내적 타당성을 높이고 두 감시체계의 특성 차이를 해석하는 근거를 제공한다.

## 결론

본 연구에서 VAE는 VAP보다 더 빈번하게 발생하였으며 감시에 소요되는 시간도 현저히 짧은 것으로 나타났다. 인공호흡기 사용기간은 두 사건에서 공통적으로 확인된 관련 요인이었으나, VAP와 VAE는 서로 다른 관련 요인 특성을 보였으며 폐렴(VAP 및 PVAP)에서는 임상적 필요에 따른 스테로이드 사용이 관련된 반면, VAE는 진정 관리와 같은 치료 과정에서 조절 가능한 요인과 관련이 있었다. 이러한 결과는 중환자실 감염 예방과 환자 안전 관리에서 VAP 감시와 VAE 감시가 목적에 따라 상호보완적인 정보를 제공할 가능성을 시사한다. 향후 다기관 연구와 COVID-19 유행 시기를 제외한 다년간 자료를 활용한 연구가 필요하며, VAE 감시를 기반으로 한 중재 효과를 평가하는 연구를 제언한다.

## Figures and Tables

**Table 1.** Incidence of Ventilator-associated Pneumonia and Ventilator-associated Events According to Intensive Care Unit Type, Season, and Duration of Mechanical Ventilation

Variables	Ventilator-days	VAP		VAE					
				VAC-Plus		IVAC-Plus		PVAP	
		Events^†^	Rate^‡^	Events^†^	Rate^‡^	Events^†^	Rate^‡^	Events^†^	Rate^‡^
Overall	10,542	31	2.9	84	8.0	38	3.6	19	1.8
Type of ICU									
ICU	6,220	15	2.4	52	8.4	23	3.7	12	1.9
NCU	2,135	11	5.2	17	8.0	6	2.8	3	1.4
CCU	1,026	3	2.9	10	9.7	6	5.8	2	1.9
CSU	1,161	2	1.7	5	4.3	3	2.6	2	1.7
Season									
Spring (Mar–May)	2,762	4	1.4	24	8.7	11	4.0	6	2.2
Summer (Jun–Aug)	2,563	8	3.1	22	8.6	8	3.1	3	1.2
Autumn (Sep–Nov)	2,652	12	4.5	28	10.6	12	4.5	7	2.6
Winter (Dec–Feb)	2,565	7	2.7	10	3.9	7	2.7	3	1.2
DMV (days)									
3–7	2,018	3	1.5	4	2.0	0	0.0	0	0.0
8–14	2,809	9	3.2	28	10.0	9	3.2	4	1.4
15–21	2,262	9	4.0	22	9.7	12	5.3	5	2.2
22–28	1,331	5	3.8	11	8.3	7	5.3	5	3.8
29–56	1,672	5	3.0	16	9.6	8	4.8	4	2.4
≥ 57	450	0	0	3	6.7	2	4.4	1	2.2

VAP = Ventilator-associated pneumonia; VAE = Ventilator-associated event; VAC = Ventilator-associated condition; IVAC = Infection-related ventilator-associated complication; PVAP = Possible ventilator-associated pneumonia; ICU = Intensive care unit; NCU = Neurological intensive care unit; CCU = Coronary care unit; CSU = Cardiocerebrovascular intensive care unit; DMV = duration of mechanical ventilation. ^†^Events indicate the number of event episodes. ^‡^Rate per 1,000 ventilator-days.

**Table 2.** Univariate Analysis of Factors Associated with the Occurrence of Ventilator-associated Pneumonia and Ventilator-associated Events (N = 942)

Variables	Study population	VAP			VAE								
					VAC-Plus			IVAC-Plus			PVAP		
		Yes (n = 31)	No (n = 911)	χ^2^*/Z*	Yes (n = 79)	No (n = 863)	χ^2^*/Z*	Yes (n = 37)	No (n = 905)	χ^2^*/Z*	Yes (n = 19)	No (n = 923)	χ^2^*/Z*
Sex				6.51^*^			0.24			3.61			0.05
Men	572 (60.7)	12 (38.7)	560 (61.5)		50 (63.3)	522 (60.5)		28 (75.7)	544 (60.1)		12 (63.2)	560 (60.7)	
Women	370 (39.3)	19 (61.3)	351 (38.5)		29 (36.7)	341 (39.5)		9 (24.3)	361 (39.9)		7 (36.8)	363 (39.3)	
Age group (years)				6.24^*^			1.57			0.30			2.72
≤ 39	39 (4.1)	4 (12.9)	35 (3.8)		5 (6.3)	34 (3.9)		2 (5.4)	37 (4.1)		2 (10.5)	37 (4.0)	
40–64	283 (30.0)	8 (25.8)	275 (30.2)		26 (32.9)	257 (29.8)		12 (32.4)	271 (29.9)		7 (36.8)	276 (29.9)	
≥ 65	620 (65.8)	19 (61.3)	601 (66.0)		48 (60.8)	572 (66.3)		23 (62.2)	597 (66.0)		10 (52.6)	610 (66.1)	
Comorbidities													
Hypertension	507 (53.8)	13 (41.9)	494 (54.2)	1.82	32 (40.5)	475 (55.0)	6.15^*^	13 (35.1)	494 (54.6)	5.41^*^	6 (31.6)	501 (54.3)	3.86^*^
Cerebrovascular disease	141 (15.0)	9 (29.0)	132 (14.5)	4.98^*^	11 (13.9)	130 (15.1)	0.07	5 (13.5)	136 (15.0)	0.06	2 (10.5)	139 (15.1)	0.30
Department				9.80^**^			10.48^**^			3.72			7.36
Medical	655 (69.5)	14 (45.2)	641 (70.4)		50 (63.3)	605 (70.1)		23 (62.2)	632 (69.8)		11 (57.9)	644 (69.8)	
Surgical	158 (16.8)	8 (25.8)	150 (16.5)		9 (11.4)	149 (17.3)		5 (13.5)	153 (16.9)		4 (21.1)	154 (16.7)	
Other	129 (13.7)	9 (29.0)	120 (13.2)		20 (25.3)	109 (12.6)		9 (24.3)	120 (13.3)		4 (21.1)	125 (13.5)	
APACHE Ⅱ score	25.0 (21.0, 29.0)	25.0 (21.0, 28.0)	25.0 (21.0, 29.0)	-0.752	25.0 (21.0, 30.0)	25.0 (21.0, 29.0)	-0.296	25.0 (21.0, 29.5)	25.0 (21.0, 29.5)	-0.06	25.0 (21.3, 27.0)	25.0 (21.0, 29.0)	-0.60
DMV (days)	8.0 (5.0, 14.3)	16.0 (10.0, 26.0)	8.0 (4.0, 14.0)	−5.12^***^	16.0 (11.0, 25.0)	7.0 (4.0, 14.0)	−8.86^***^	21.0 (14.5, 31.0)	8.0 (4.0, 14.0)	−7.09^***^	23.0 (15.0, 32.0)	8.0 (4.0, 14.0)	−5.24^***^
3–7	439 (46.6)	3 (9.7)	436 (47.9)	29.67^***^	4 (5.1)	435 (50.4)	82.44^***^	0 (0.0)	439 (48.5)	71.87^***^	0 (0.0)	439 (47.6)	49.83^***^
8–14	268 (28.5)	9 (29.0)	259 (28.4)		28 (35.4)	240 (27.8)		9 (24.3)	259 (28.6)		4 (21.1)	264 (28.6)	
15–21	130 (13.8)	9 (29.0)	121 (13.3)		22 (27.8)	108 (12.5)		12 (32.4)	118 (13.0)		4 (21.1)	126 (13.7)	
22–28	54 (5.7)	5 (16.1)	49 (5.4)		10 (12.7)	44 (5.1)		6 (16.2)	48 (5.3)		5 (26.3)	49 (5.3)	
29–56	45 (4.8)	5 (16.1)	40 (4.4)		13 (16.5)	32 (3.7)		8 (21.6)	37 (4.1)		5 (26.3)	40 (4.3)	
≥ 57	6 (0.6)	0 (0.0)	6 (0.7)		2 (2.5)	4 (0.5)		2 (5.4)	4 (0.4)		1 (5.3)	5 (0.5)	
EN	526 (55.8)	20 (64.5)	506 (55.5)	0.98	47 (59.5)	479 (55.5)	0.47	21 (56.8)	505 (55.8)	0.01	9 (47.4)	517 (56.0)	0.56
IV steroid use	419 (44.5)	21 (67.7)	398 (43.7)	7.02^**^	45 (57.0)	374 (43.3)	5.45^*^	22 (59.5)	397 (43.9)	3.50	15 (78.9)	404 (43.8)	9.32^**^
DS (%)	80.0 (29.3, 100.0)	90.6 (44.4, 100.0)	80.0 (28.6, 100.0)	−1.05	94.1 (70.6, 100.0)	78.9 (25.9, 100.0)	−2.83^**^	93.5 (67.9, 100.0)	80.0 (28.4, 100.0)	−1.82	97.5 (82.7, 100.0)	80.0 (28.6, 100.0)	−2.41^*^
First 3 days	55.6 (11.1, 100.0)	100.0 (44.4, 100.0)	55.6 (11.1, 100.0)	−2.75^**^	88.9 (44.4, 100.0)	44.4 (0.0, 100.0)	−5.10^***^	100.0 (66.7, 100.0)	55.6 (5.6, 100.0)	−4.32^***^	100.0 (66.7, 100.0)	55.6 (11.1, 100.0)	−3.64^***^
First 7 days	28.6 (4.8, 61.6)	52.4 (19.0, 100.0)	28.6 (4.8, 61.9)	−2.71^**^	85.7 (42.9, 100.0)	23.8 (4.8, 57.1)	−7.91^***^	90.5 (64.3, 100.0)	23.8 (4.8, 57.1)	−6.84^***^	100.0 (76.2, 100.0)	23.8 (4.8, 61.9)	−5.71^***^
% of ICU days	21.4 (7.1, 44.4)	30.41 (16.7, 50.0)	21.4 (6.7, 44.4)	−1.23	42.9 (18.2, 69.2)	20.0 (6.3, 42.3)	−5.04^***^	54.5 (23.3, 70.6)	20.0 (6.5, 42.9)	−4.52^***^	56.3 (29.7, 69.4)	20.0 (6.7, 44.0)	−4.21^***^
LOS _ICU_ (days)	14.0 (8.0, 22.0)	21.0 (14.0, 28.0)	14.0 (8.0, 21.0)	−3.67^***^	21.0 (15.0, 29.0)	13.0 (8.0, 20.0)	−6.07^***^	23.0 (15.0, 32.5)	13.0 (8.0, 21.0)	−4.95^***^	24.5 (15.5, 33.3)	14.0 (8.0, 21.0)	−3.52^***^

Categorical variables are presented as n (%), continuous variables are presented as median (interquartile range [IQR)] and compared using the Mann–Whitney U test. VAP = Ventilator-associated pneumonia; VAE = Ventilator-associated event; VAC = Ventilator-associated condition; IVAC = Infection-related ventilator-associated complication; PVAP = Possible ventilator-associated pneumonia; APACHEⅡ = Acute physiology and chronic health evaluation II; DMV = Duration of mechanical ventilation; EN = Early nutritional support; IV = Intravenous; DS = Deep sedation; LOS = Length of stay; ICU = Intensive care unit. Bold values indicate *p* < .01 for the variables of primary interest included in the multivariable logistic regression models, excluding covariates. Values in the “Yes” columns represent the number of patients with at least one event, not the total number of event episodes. Only the first event per patient was included in the analysis of associated factors.

* *p* < .05,

** *p* < .01,

*** *p* < .001.

**Table 3.** Multivariable Logistic Regression Analysis of Factors Associated with Ventilator-associated Pneumonia and Ventilator-associated Event Categories

Variables	VAP				VAE											
					VAC-Plus				IVAC-Plus				PVAP			
	B	OR	95% CI	*p*	B	OR	95% CI	*p*	B	OR	95% CI	*p*	B	OR	95% CI	*p*
Sex^†^																
Men	−0.97	0.38	0.18–0.82	.014	−0.06	0.94	0.56–1.59	.827	0.67	1.96	0.84–4.58	.119	−0.04	0.96	0.33–2.76	.940
Age group (years)^†^																
40–64	−1.27	0.28	0.07–1.11	.071	−0.39	0.68	0.22–2.06	.490	−0.48	0.62	0.12–3.19	.567	−1.17	0.31	0.05–1.92	.208
≥ 65	−0.92	0.40	0.11–1.45	.162	−0.34	0.72	0.24–2.10	.541	−0.11	0.89	0.18–4.35	.890	−1.20	0.30	0.05–1.83	.193
Comorbidities^†^																
Hypertension^†^	−0.42	0.66	0.30–1.46	.301	−0.44	0.64	0.38–1.08	.095	−0.60	0.55	0.26–1.17	.120	−0.51	0.60	0.20–1.77	.355
Cerebrovascular disease^†^	0.92	2.51	0.97–6.54	.059	0.13	1.14	0.52–2.50	.750	0.35	1.42	0.44–4.53	.557	0.28	1.33	0.24–7.39	.745
Department^†^																
Medical	−0.99	0.37	0.15–0.96	.040	−0.60	0.55	0.29–1.03	.063	−0.48	0.62	0.25–1.56	.309	−0.38	0.69	0.19–2.46	.561
Surgical	0.12	1.13	0.38–3.31	.827	−0.74	0.48	0.19–1.19	.111	−0.29	0.75	0.22–2.62	.654	0.85	2.34	0.48–11.28	.291
IV steroid use^†^	1.16	3.19	1.34–7.59	.009	0.04	1.04	0.61–1.78	.892	0.13	1.14	0.52–2.51	.739	1.42	4.14	1.14–15.09	.031
DMV (days)	0.04	1.04	1.02–1.07	< .001	0.07	1.07	1.05–1.09	< .001	0.07	1.07	1.05–1.10	< .001	0.07	1.07	1.04–1.10	< .001
DS (% of ICU days)	0.01	1.01	0.99–1.02	.347	0.02	1.02	1.01–1.03	< .001	0.03	1.03	1.01–1.04	< .001	0.03	1.03	1.02–1.05	< .001
Constant	-2.84	0.06		.001	−3.00	0.05		< .001	−5.07	0.01		< .001	−5.96	0.00		< .001
Model ꭓ^2^ (df = 10)(*p*)	42.55 (< .001)				84.86 (< .001)				65.55 (.001)				47.00 (.001)			
-2 Log Likelihood	230.09				457.94				246.52				138.95			
Nagelkerke R^2^	.18				.20				.24				.27			
Hosmer–Lemeshow χ² (df = 8)(*p*)	5.68 (.683)				13.50 (.096)				5.34 (.721)				3.71 (.883)			

Models were adjusted for sex, age group, hypertension, cerebrovascular disease, and department. VAP = Ventilator-associated pneumonia; VAE = Ventilator-associated event; VAC = Ventilator-associated condition; IVAC = Infection-related ventilator-associated complication; PVAP = Possible ventilator-associated pneumonia; OR = Odds ratio; CI = Confidence interval; IV = Intravenous; DMV = Duration of mechanical ventilation; DS = Deep sedation; ICU = Intensive care unit; df = Degrees of freedom. ^†^Indicates dummy variables. Reference categories (variable) = women (sex); < 39 years (age group); no comorbidity (comorbidities); other (department); no IV steroid use (IV steroid use).
